# Notch and Delta are required for survival of the germline stem cell lineage in testes of *Drosophila melanogaster*

**DOI:** 10.1371/journal.pone.0222471

**Published:** 2019-09-12

**Authors:** Chun L. Ng, Yue Qian, Cordula Schulz

**Affiliations:** 1 University of Texas Southwestern Medical Center, Dallas, Texas, United States of America; 2 University of North Georgia, Department of Biology, Oakwood, Georgia, United States of America; 3 University of Georgia, Department of Cellular Biology, Athens, Georgia, United States of America; National Cancer Institute, UNITED STATES

## Abstract

In all metazoan species, sperm is produced from germline stem cells. These self-renew and produce daughter cells that amplify and differentiate dependent on interactions with somatic support cells. In the male gonad of *Drosophila melanogaster*, the germline and somatic cyst cells co-differentiate as cysts, an arrangement in which the germline is completely enclosed by cytoplasmic extensions from the cyst cells. Notch is a developmentally relevant receptor in a pathway requiring immediate proximity with the signal sending cell. Here, we show that Notch is expressed in the cyst cells of *wild-type* testes. Notch becomes activated in the transition zone, an apical area of the testes in which the cyst cells express stage-specific transcription factors and the enclosed germline finalizes transit-amplifying divisions. Reducing the ligand Delta from the germline cells via RNA-Interference or reducing the receptor Notch from the cyst cells via CRISPR resulted in cell death concomitant with loss of germline cells from the transition zone. This shows that Notch signaling is essential for the survival of the germline stem cell lineage.

## Introduction

The Notch signaling pathway is highly conserved and plays versatile roles in development, such as nervous system formation, cardiac patterning, and sensory hair formation [1–3]. In many developmental contexts Notch specifies cell fate decisions. In the developing vertebrate eye, for example, Notch regulates which cells develop into glial cells and which develop into optic neurons [4,5]. During *C*. *elegans* vulva development, Notch prevents nearby cells from becoming central vulval cells [6]. In other developmental contexts Notch regulates the survival of cells. In the murine nervous system, loss of Notch results in the death of progenitor cells and newly differentiated cells [7]. Notch signaling has also been associated with cell survival in B-cell malignancies, prostate cancer cells, and myeloma cells [8–10].

**Fig 1:**
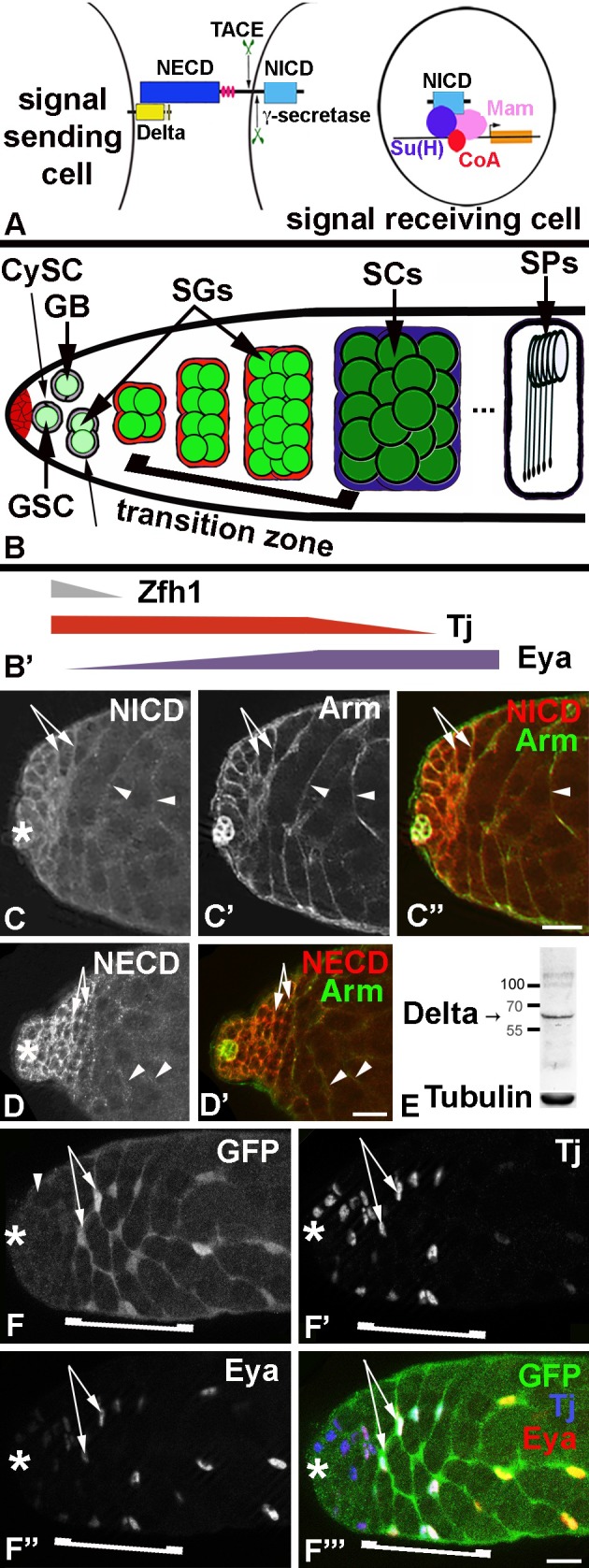
Notch signaling was activated in the transition zone. A) Cartoon depicting the canonical Notch signaling pathway. NECD: Notch extra-cellular domain, NICD: Notch intra-cellular domain, Su(H): Suppressor of Hairless, Mam: Mastermind, CoA: transcriptional co-regulator, TACE and γ-secretase: proteases cleaving the Notch receptor.B) Cartoon of spermatogenesis with a focus on the apical region. GSC: germline stem cell, GB: gonialblast; SGs: spermatogonia; SCs: spermatocytes; SPs: spermatids; CySC: Cyst Stem Cell, CCs: cyst cells, bracket: transition zone. B’) Arrows represent the regions of the testis in which indicated transcription factors are expressed in the CySC and cyst cells. Color-coding corresponds to the colors in B). C-F”’) Asterisks mark the apical tips of the testes, scale bars: 30 μm. C-D‘’) Apical region of a *wt* testis showing the expression of C) NICD, D) NECD, C’) Arm, and C”, D’) their co-localization on the cyst cell membranes. Arrows and arrowheads point to cyst cells surrounding spermatogonia and spermatocytes, respectively, n>50. E) Western blot probed with anti-Dl and anti-Tubulin antibodies, as indicated. F-F”’) The F) NRE-eGFP reporter for Notch activation co-localized with F’, F”’) Tj and F”, F”’) Eya within the cyst cell nuclei of a *wt* testis (n>50). The transition zone is depicted by a bracket.

The canonical Notch signaling pathway is rather simple. Upon activation, the Notch receptor is proteolytically cleaved causing the release of the intra-cellular portion of the protein, called the Notch intra-cellular domain (NICD). The NICD enters the nucleus and joins a protein complex bound to chromatin altering the transcription of target genes. This complex includes the transcription factors Suppressor of Hairless (Su(H)) in *Drosophila melanogaster* and Mastermind, as well as other potential co-regulators (Fig 1A). Additional levels of regulation are added to the pathway via receptor-ligand internalization, post-translational modification, and protein stability [11, 12].

While vertebrates have several Notch receptors and ligands, the *Drosophila* genome only contains one Notch receptor and two ligands, Delta (Dl) and Serrate. Both, the receptor and the ligands are transmembrane proteins [13]. Thus, in order for Notch signaling to occur the ligand-expressing cells have to be in intimate contact with the receptor-expressing cells. Such a cellular architecture is found in spermatogenesis where germline cells are associated with and surrounded by somatic support cells [14, 15]. *Drosophila* testes contain Germline Stem Cells (GSCs) that produce sperm cells and Cyst Stem Cells (CySCs) that produce somatic support cells, the cyst cells. Both stem cell populations are arranged around a group of somatic hub cells at the apical tip of the testis (Fig 1B). GSCs and CySCs undergo constant cell divisions that result in asymmetric outcomes, the stem cell daughters that remain attached to the hub cells become new stem cells, while the stem cell daughters that are displaced away from the hub become gonialblasts and cyst cells, respectively [14, 16]. One gonialblast and two cyst cells form a cyst. During this process, the cyst cells grow cytoplasmic extensions around the gonialblast to fully enclose it [17, 18]. The two cyst cells serve as an adhesion and signaling center for the developing germline cells. Accordingly, defects in cyst formation result in abnormal proliferation and differentiation of the germline and cyst cells [17–21].

During subsequent cyst differentiation, the gonialblast engages in transit amplifying mitotic divisions to produce increasing numbers of early stage germline cells, called spermatogonia. The cysts with the gonialblasts and the spermatogonia are located basal to the stem cells within the apical region of the testes. After ceasing amplifying divisions, the germline cells become spermatocytes, grow in size, and reduce their chromosomal content by meiosis. Once haploid, the germline cells compact their DNA and change their morphology from round to elongated cells, thereby becoming spermatids (Fig 1B). Fully developed sperm is found at the basal end of the testes [22].

The two cyst cells surrounding each cluster of developing germline cells differentiate as well, as evident by their cellular growth and the expression of different sets of molecular markers [23]. CySCs express the transcription factor Zinc Finger Homeodomain-1 (Zfh-1) [24]. The expression level of Zfh-1 drops in the CySC daughters as they differentiate and become displaced away from the stem cell area (Fig 1B and 1B’). CySCs also express the transcription factor Traffic Jam (Tj). Tj expression is maintained in cyst cells surrounding gonialblasts and spermatogonia (Fig 1B and 1B’) but drops in expression level in cyst cells that enclose spermatocytes [25]. Another transcription factor, Eyes absent (Eya), is expressed at low levels in CySCs, at higher levels in cyst cells surrounding spermatogonia, and at highest levels in cyst cells surrounding spermatocytes (Fig 1B and 1B’) [26]. The apical region of the testes in which Tj expression is high and Eya expression is increasing within the cyst cells coincides with the positions in which spermatogonia have reached the final two rounds of transit amplifying divisions and develop into spermatocytes. This area is, in the following, referred to as the transition zone (bracket in Fig 1B). Here, we show that Notch is expressed and activated in the cyst cells of the transition zone. We present evidence that the ligand Dl is required within the germline cells and that the receptor Notch is required within the cyst cells for the survival of the germline stem cell lineage.

## Materials and methods

### Fly husbandry

Flies were maintained on standard cornmeal/molasses diet at room temperature. *tj-gal4* was obtained from the Kyoto stock center and all other stocks from the Bloomington *Drosophila* Stock Center (BDSC; genotypes and stock numbers are listed in Table 1).

**Table 1:** Tools for studying Notch signaling.

BL#	Description	Genotype	Phenotype Observed
29625	UAS-*Cut*-*i*	*y[1] v[1]; P{y[+t7*.*7] v[+t1*.*8] = TRiP*.*JF03304}attP2*	No
33967	UAS-*Cut*-*i*	y[1] sc[*] v[1]; P{y[+t7.*7] v[+t1*.*8] = TRiP*.*HMS00924}attP2*	No
8611	UAS-*Delta* with GFP inserted in extracellular domain	w[*]; P{w[+mC] = UAS-Dl::*GFP}DA55*	No
9319	UAS-*Delta*	y1 w*; P{UAS-dl.*H}2*	No
26697	UAS-dominant negative *Delta*	w*; P{UAS-Dl.*DN}TJ2/CyO*	No
26698	UAS-dominant negative *Delta*	w*; P{UAS-Dl.*DN}TJ3*	No
28032	UAS-*Delta*-*i*	*y1 v1; P{TRiP*.*JF02867}attP2*	No
34322	UAS-*Delta*-*i*	y1 sc* v1; P{TRiP.*HMS01309}attP2*	Yes
36784	UAS-*Delta*-*i*	y1 sc* v1; P{TRiP.*GL00520}attP40*	No
26322	UAS-*Enhancer of split-m8-i*	*y[1] v[1]; P{y[+t7*.*7] v[+t1*.*8] = TRiP*.*JF02096}attP2*	No
26675	UAS-*Enhancer of split-mbeta*	y[1] w[*]; P{w[+mC] = UAS-E(spl)mbeta}48.*1*	No
26679	UAS-*Enhancer of split-m4*	w[*]; P{w[+mC] = UAS-E(spl)m4-BFM.*A}15*.*5*	No
26872	UAS-*Enhancer of split*	w*; P{UAS-E(spl)m8-HLH.*T}3*	No
27179	UAS-*E(spl)-malpha*	y[1] w[*]; P{w[+mC] = UAS-E(spl)malpha-BFM.*A}A17*.*4*	No
28735	UAS-*Hintsight*-*i*	*y[1] v[1]; P{y[+t7*.*7] v[+t1*.*8] = TRiP*.*JF03162}attP2*	No
33901	UAS-*Hintsight*-*i*	y[1] sc[*] v[1]; P{y[+t7.*7] v[+t1*.*8] = TRiP*.*HMS00843}attP2*	No
33943	UAS-*Hintsight*-*i*	y[1] sc[*] v[1]; P{y[+t7.*7] v[+t1*.*8] = TRiP*.*HMS00894}attP2/TM3*, *Sb[1]*	No
6578	UAS-dominant negative *Kuzbanian*	w[*]; P{w[+mC] = UAS-kuz.*DN}2*	No
5830	UAS-constitutively active *Notch*	y1 w*; P{UAS-Dl::*N*.*ΔECN}B2a2*	Yes
m7077	UAS-*Notch*-*i*	w*; P{UAS-N.*dsRNA*.*P}9G*	No
7078	UAS-*Notch*-*i*	*P{UAS-N*.*dsRNA*.*P}14E*, w*	No
26820	UAS-*Notch*	w*; P{UAS-Nfull}3	No
29856	UAS-*Notch* truncated, ligand non-responsive	*P{w[+mC] = UAS-N*.*DeltaEGF*.*LV}1*, *w[1118]*	No
27988	UAS-*Notch*-*i*	*y[1] v[1]; P{y[+t7*.*7] v[+t1*.*8] = TRiP*.*JF02959}attP2*	No
28981	UAS-*Notch*-*i*	*y[1] v[1]; P{y[+t7*.*7] v[+t1*.*8] = TRiP*.*JF01637}attP2*	No
31180	UAS-*Notch*-*i*	*y[1] v[1]; P{y[+t7*.*7] v[+t1*.*8] = TRiP*.*JF01693}attP2*	No
31502	UAS-*Notch*-*i*	*y[1] v[1]; P{y[+t7*.*7] v[+t1*.*8] = TRiP*.*JF01043}attP2*	No
31503	UAS*-Notch*-*i*	*y[1] v[1]; P{y[+t7*.*7] v[+t1*.*8] = TRiP*.*JF01053}attP2*	No
33616	UAS-*Notch*-*i*	*y1 v1; P{TRiP*.*HMS00009}attP2*	No
35640	UAS-*Notch*-*i*	y[1] sc[*] v[1]; P{y[+t7.*7] v[+t1*.*8] = TRiP*.*GLV21004}attP2*	No
36784	UAS-*Notch*-*i*	y[1] sc[*] v[1]; P{y[+t7.*7] v[+t1*.*8] = TRiP*.*GL00520}attP40*	No
31182	UAS-*Numb*-*i*	*y[1] v[1]; P{y[+t7*.*7] v[+t1*.*8] = TRiP*.*JF01695}attP2*	No
35045	UAS-*Numb*-*i*	y[1] sc[*] v[1]; P{y[+t7.*7] v[+t1*.*8] = TRiP*.*HMS01459}attP2*	No
28046	UAS-*mastermind*-*i*	*y[1] v[1]; P{y[+t7*.*7] v[+t1*.*8] = TRiP*.*JF02881}attP2*	No
26023	UAS-*neuralized*-*i*	*y[1] v[1]; P{y[+t7*.*7] v[+t1*.*8] = TRiP*.*JF02048}attP2*	No
35412	UAS-*neuralized*-*i*	y[1] sc[*] v[1]; P{y[+t7.*7] v[+t1*.*8] = TRiP*.*GL00334}attP2*	No
27498	UAS-*Nicastrin*-*i*	*y[1] v[1]; P{y[+t7*.*7] v[+t1*.*8] = TRiP*.*JF02648}attP2*	No
27681	UAS-*Presenilin*-*i*	*y[1] v[1]; P{y[+t7*.*7] v[+t1*.*8] = TRiP*.*JF02760}attP2/TM3*, *Sb[1]*	No
5815	UAS-*Serrate*	w[*]; P{w[+mC] = UAS-Ser.*mg5603}SS1*	No
28713	UAS-*Serrate*-*i*	*y[1] v[1]; P{y[+t7*.*7] v[+t1*.*8] = TRiP*.*JF03140}attP2*	No
34700	UAS-*Serrate*-*i*	y[1] sc[*] v[1]; P{y[+t7.*7] v[+t1*.*8] = TRiP*.*HMS01179}attP2*	No
28900	UAS-*Su(H)*–*i*	*y[1] v[1]; P{y[+t7*.*7] v[+t1*.*8] = TRiP*.*HM05110}attP2/TM3*, *Sb[1]*	No
30727	Expresses eGFP under control of NRE	w[1118]; P{w[+m*] = NRE-EGFP.*S}5A*	N/A
30728	Expresses eGFP under control of NRE	w[1118]; P{w[+m*] = NRE-EGFP.*S}1*	N/A

Fly stocks used to study Notch signaling in adult testes and their description, as indicated;

*: unspecified allele.

### Immunofluorescence and image analysis

Testes were isolated as previously described [27]. The mouse anti-Eyes absent (Eya) antibody (1:10) developed by S. Benzer and N. M. Bonini, the mouse anti-Dl antibody developed by S. Artavanis-Tsakonis, and the rat anti-Vasa antibody (1:10) developed by A. C. Spradling and D. Williams were obtained from the Developmental Studies Hybridoma Bank (DSHB), created by the NICHD of the NIH and maintained at The University of Iowa, Department of Biology, Iowa City, IA 52242. Goat anti-Armadillo antibody (1:500) was obtained from Santa Cruz Biotechnology Inc. (sc28653), and the rabbit anti-GFP antibody (1:200) was obtained from Life Technologies (A11122), Guinea pig-anti-Traffic Jam antibody (1:5000) was a gift from Dorothea Godt. Secondary Alexa 488, 568, and 647-coupled antibodies (1:1000) and Slow Fade Gold embedding medium were obtained from Life Technologies. Images were taken using a Zeiss Axiophot with a digital camera and apotome and processed using Axiovision Rel. software. Images were analyzed using ImageJ and processed with Photoshop.

### Statistics

Statistical relevance was determined using the Graphpad student’s t-test.

### SDS page and western blots

SDS page and western blotting was performed following standard procedures. The membranes were blocked in 1x TBS-T (5% BSA in Tris-buffered saline and 0.1% Tween 20) at 4°C overnight. Membranes were incubated with primary mouse anti-Dl (1:100; DSHB C594.9B) at 4°C overnight. Primary antibody was detected by peroxidase conjugated anti-mouse antibody (1:10,000; Sigma Aldrich). Proteins were visualized using Amersham ECL Prime Western Blotting Detection Reagent (GE Healthcare).

### Flp/FRT-recombination

FLP/FRT lines, FRT82B *Dl*^*RevF10*^, *Ser*^*Rx106*^ and FRT82B^*rosy*^, were crossed to hs-flp; FRT82B-GFP and progeny raised at 25°C. One to three-day-old adult male flies were heat shocked for one hour at 37°C for three consecutive days and testes were dissected seven days after heat shock.

### UAS/Gal4-expression studies and CRISPR

gRNA constructs were injected into into *w*^1118^ flies by the company The Best Gene. The stocks were crossed with UAS-Cas9 and the progeny mated with Gal4 transactivators to specifically express N CRISPR in the germline or the soma of the testes in the next generation. Fly crosses were set up at 18°C and progeny shifted as larvae or adults to the restrictive temperatures as described in the result and discussion section of the manuscript.

### Apoptosis detection

Cells in apoptosis were detected using terminal deoxynucleotidyl transferase dUTP nick end labeling (TUNEL). The Apoptag Red In Situ Kit was obtained from Millipore (S1765) and tissue was treated according to the manufacturer’s instructions.

## Results and discussion

### The Notch pathway is activated in the transition zone of *wild-type* testes

To explore if Notch signaling plays a role in germline-soma interactions, we first investigated the expression pattern of Notch in *wild-type* (*wt*) testes. For this, we employed two commonly used Notch antibodies: one directed against the NICD and the other against the Notch extra-cellular domain (NECD). Both antibodies were detected in the cyst cells surrounding the germline cells. To confirm expression in the cyst cells, we co-labeled the testes with the marker anti-Armadillo (Arm) that has previously been shown to localize to the membranes of the cyst cells, resulting in a net-like pattern of expression [17, 18]. NICD (Fig 1C) and NECD (Fig 1D) were detectable in a similar net-like pattern both in the apical region where the cyst cells surround the spermatogonia (Fig 1C and 1D arrows) and more basally where cyst cells surround the spermatocytes (Fig 1C and 1D arrowheads). This expression pattern overlapped with the pattern produced by the Arm staining (Fig 1C’, 1C” and 1D’). We were not able to detect the expression of Dl by immuno-fluorescence staining of testes. However, western blots of testes revealed a band at the predicted size of about 62 kDa (Figs 1E and S1), showing that Dl is expressed in the male gonad.

To confirm that Notch is active within the cyst cells and to address at which stage of cyst development Notch is activated, we used readouts for Notch stimulation. A Notch reporter, Notch Response Element-e-Green Fluorescent Protein (NRE-eGFP), consists of the GFP coding region under the control of Su(H) binding sites. Without Notch signaling, no GFP is expressed from the NRE-eGFP. When Notch is stimulated, Su(H), in combination with NICD, acts as an activator and GFP expression from NRE-eGFP is apparent [28]. Su(H)-driven GFP was not detectable above background in the cyst cells near the apical tip (Fig 1F, arrowhead). Instead, we detected strong GFP-signal in cyst cells starting more basally within the apical region (Fig 1F, arrows). Co-localization experiments revealed that NRE-eGFP is detectable in cyst cells of the apical region that also express Tj and Eya (Fig 1F”–1F”’, arrows). Though we cannot exclude the possibility that the expression of the NRE-eGFP at the tip of the testis is below detection level, our observations suggest that Notch most likely becomes first activated in the cyst cells of the transition zone (bracket in Fig 1B and 1F–F”’).

### Over-activation of Notch in the cyst cells of epidermal growth factor (EGF) mutant testes had a drastic effect on germline development

The Notch signaling pathway has been implicated in the specification of the male germline stem cell niche in *Drosophil*a males [29]. However, a role for Notch in the adult testes remained elusive, most likely because viable, temperature-sensitive alleles of Notch and many of the other tools (Table 1) that can be used to study Notch signaling in other tissues did not display a testis phenotype. This suggests that either loss of Notch has no effect on testes, or that it is extremely hard to eliminate Notch signaling from testes. Based on the above expression study, it appears that the area in which we first detect Notch activation is the same region of the testes in which EGF signaling is active. The EGF signaling pathway plays a major role in germline-soma interactions. First, signaling via EGF from the germline cells to the EGF-receptor (EGFR) on the cyst cells instructs cyst formation [17, 18]. Subsequently, EGF signaling regulates cyst development. While a low dose of EGF from the germline to the cyst cells is required for the germline cells to progress through spermatogonial stages, a high dose of EGF signaling appears to promote the transition from early stage cysts containing spermatogonia into late stage cysts containing spermatocytes [20, 30]. A temperature-sensitive allele of EGF, *spitz*^*77-20*^, has served as an excellent tool for gaining more insight into spermatogenesis. Previous research has established that the *spitz*^*77-20*^ mutant testis phenotype from animals held at an intermediate temperature of 26.5°C could easily be modified by additional mutations in pathways regulating cyst development [18, 31]. Thus, prior to employing further tools for eliminating Notch signaling from otherwise *wt* testes we first utilized the *spitz*^*77-20*^ allele to investigate for a potential genetic interaction.

**Fig 2:**
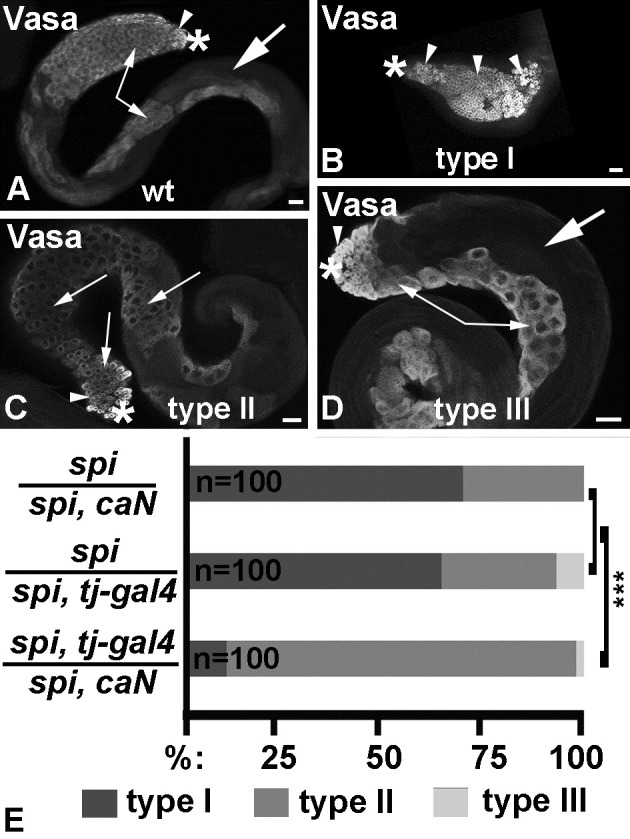
Activation of Notch within the cyst cells of EGF mutant testes modified the phenotype. A-D) Whole testes stained for the germline marker, Vasa. Asterisks mark the apical tips of the testes, arrowheads point to spermatogonia, small arrow point to spermatocytes, large arrows point to spermatids, scale bars: 30 μm. A) *wt*, B) *spi* type I, C) *spi* type II, and D) *spi* type III testis. E) Bar graph demonstrating the percentage of type I, II, and III testes in different genetic backgrounds (as indicated). ***: P-value below 0.001.

In a *wt* testis, the germline cells vary in sizes and shapes and are easily recognizable by these characteristics. The GSCs and their transit amplifying daughters are small cells located within the apical region of the testis (Fig 2A, arrowhead), based on staining with the germline marker anti-Vasa. The spermatocytes are larger and found basal to the apical region and along the testis coil (Fig 2A, small arrows). Sperm is found at the testis base but also reaches into the lumen of the testis (Fig 2A, large arrow). We previously showed that when *spitz*^*77-20*^ mutant animals are raised at 26.5°C, the majority of the testes are tiny and contain mostly germline cells at the spermatogonia stage. These testes were classified as type I testes. Some testes are longer and also contain spermatocytes (type II testes) and/or spermatids (type III testes) [31]. As most germline cells in *spitz*^*77-20*^ mutant testes are not enclosed by cyst cells, signaling between these two cell types via Notch and its transmembrane ligand seems unlikely. With this rationale, we employed the UAS/Gal4-expression system to express a ligand-independent and constitutively active version of Notch, UAS-*Dl*::*N*.*ΔECN* (*caN*), within the cyst cells of *spitz*^*77-20*^ mutant testes [32, 33]. This version of Notch contains the yeast Upstream Activating Sequences (UAS) as the promotor upstream of a fusion between the *dl* start and membrane transport signal sequence, and the coding region of the Notch transmembrane and intracellular domains [34].

To express *caN* within the cyst cells of the testes, we used a well-established soma-Gal4 transactivator, *traffic jam-gal4* (*tj-gal4*) [35]. As expected, control animals (*spi/spi*, *caN* and *spi/spi*, *tj-gal4*) had mostly type I testes (Fig 2E) that were filled with spermatogonia (arrowheads in Fig 2B). Upon expression of *caN* in the cyst cells of *spitz*^*77-20*^ animals, we detected a drastic modification of the mutant phenotype. Almost all of the experimental testes (*spi*, *tj-gal4/spi*, *caN*) were type II testes (Fig 2E) and contained mostly spermatocytes (small arrows in Fig 2C). A few testes were of type III (Fig 2E) and contained sperm bundles (large arrow in Fig 2D). We are yet to explore how expression of *caN* allows the cells in *spitz*^*77-20*^ testes to develop past the spermatogonial stage. However, this finding suggests that Notch does play a role in cyst development, likely by promoting germline differentiation, and encouraged us to further explore the tools for generating a Notch loss-of-function phenotype.

### Reduction of Dl from the germline caused massive loss of germline cells

**Fig 3:**
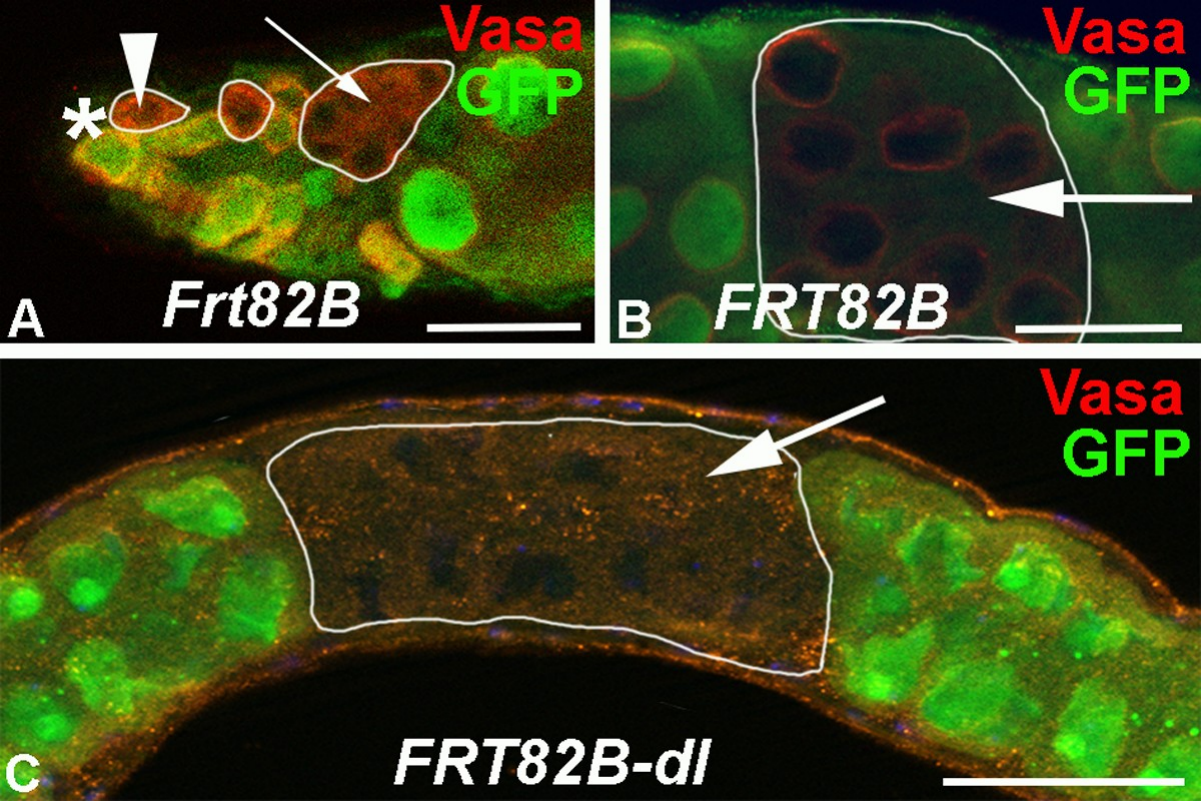
FRT/Flp-recombination produced clusters of GFP-negative germline cells. A-C) Scale bars: 50 μm, stainings as indicated, arrowhead points to a GFP-negative GSC, small arrow points to a cluster of GFP-negative spermatogonia, large arrows point to clusters of GFP-negative spermatocytes. Clones are also outlined by white circles. A) The apical tip of a control, FRT82B, testis containing three clusters of GFP-negative cells. B, C) Single GFP-negative clusters of spermatocytes along the testis coil of B) FRT82B and C) FRT82B, *dl* animals.

The expression pattern of Notch and Notch reporters suggests that the cyst cells receive the ligand from the germline. As *dl* mutation are embryonic lethal, we used the FRT/Flp-recombination technique to generate clusters of *dl* mutant germline cells in adult testes that were negatively marked by Green Fluorescent Protein (GFP) [36]. In the control animals, 18 out of 46 testes contained one or more GFP-negative clusters of germline cells (Fig 3A and 3B and Table 2). In the experimental animals, only three out of 80 testes showed a single cluster of GFP-negative germline cells (Fig 3C and Table 2). This suggests that *dl* mutant germline clones are either rarely formed or rarely survive.

**Table 2:** *dl* mutant germline clones were rarely detected in FRT/Flp clonal analysis.

Genotype	n	0	1	3	4	5 or more
Frt82B	46	28	3	1	3	11
Frt82B-*dl*	80	77	3	0	0	0

n: number of testes, numbers of GFP-negative germline clusters per testis as indicated.

**Fig 4:**
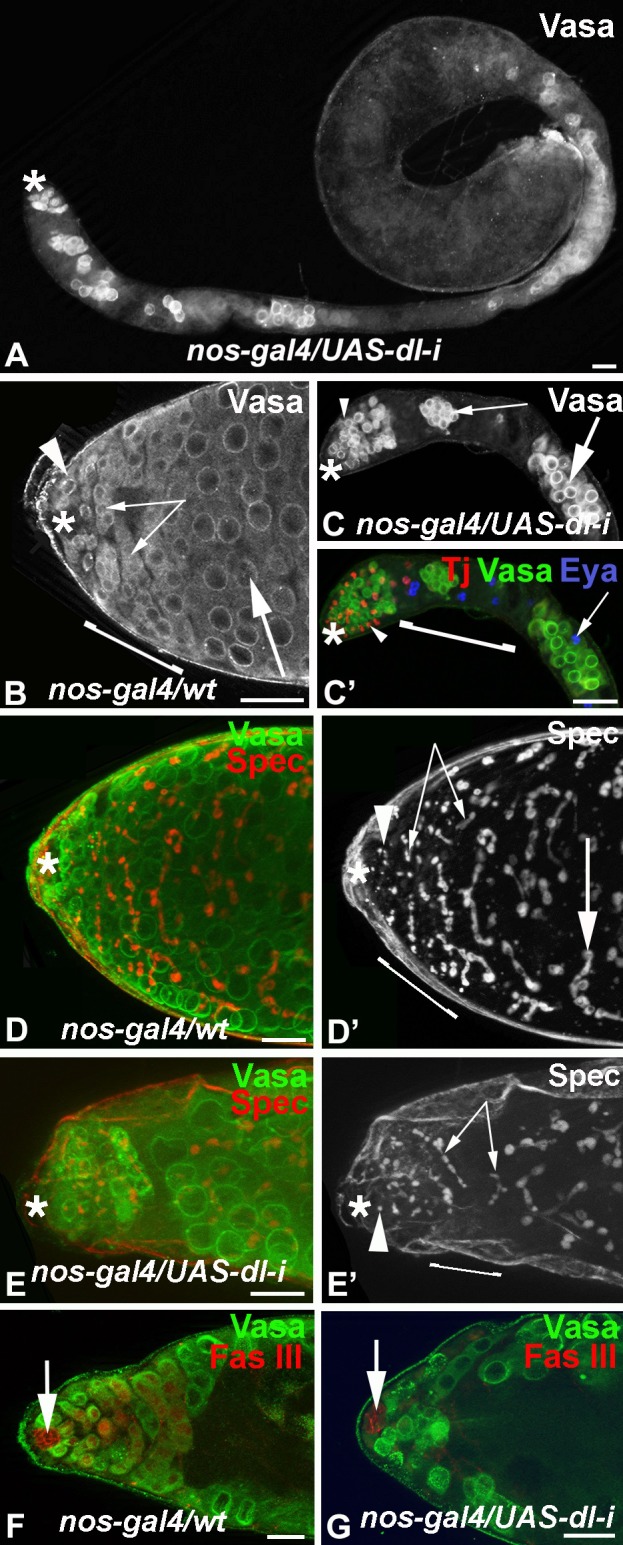
RNA*-i* against *dl* in the germline caused severe germline loss. A-G) Scale bars: 30 μm, asterisks mark the apical tips of the testes, brackets: transition zones, stainings as indicated. A) A testis from a *nos-gal4/UAS-dl-i* male eight days after the shift to the restrictive temperature, n>50. B, C) The apical testis regions of B) a *nos-gal4/wt* (n>50) and C) a *nos-gal4/UAS-dl-i* animal (n>50). Arrowheads point to GSCs and/or their immediate daughters, small arrows point to spermatogonia, large arrows point to spermatocytes. C’) Same apical testis regions as in C) but co-labeled for cyst cell markers. Arrowhead points to a Tj-positive cyst cell nucleus, arrow points to an Eya-positive cyst cell nucleus. D-E’) The apical testis regions from D, D’) a *nos-gal4/wt* (n>50) and E-E’) a *nos-gal4/UAS-dl-i* animal (n>50) showing spectrosomes (arrowheads) and fusomes (arrows). F, G) The apical testis regions of F) a *nos-gal4/wt* (n>50) and G) a *nos-gal4/UAS-dl-i* (n>50) animal showing the presence of hub cells (arrows).

To obtain higher numbers of *dl* mutant cells, we used a RNA-Interference (RNA*-i*) construct for *dl* (*UAS-dl-i*) driven either in cyst cells via *tj-gal4*, or within the germline via the *nanos-gal4* transactivator (*nos-gal4*) [37]. While expression of UAS-*dl-i* via *tj-gal4* had no morphological effect (n>50), expression of UAS-*dl-i* via *nos-gal4* caused a drastic loss of germline cells. When *nos-gal4/UAS-dl-i* animals were kept at the permissive temperature of 18°C, no effect on the testes was observed (n>50). After shifting the animals to the restrictive temperature of 29°C for eight days, testes were long and thin and contained only few germline cells (Fig 4A) while testes from control animals (*nos-gal4/wt* and *UAS-dl-i/wt*) treated under the same conditions appeared normal and contained all stages of germline cells (Fig 4B). Specifically, GSCs (arrowhead in Fig 4B), spermatogonia (small arrows in Fig 4B) and spermatocytes (large arrow in Fig 4B) filled the apical region of the testes from *nos-gal4/wt* animals.

By eight days after the temperature shift to 29°C, the testes from *nos-gal4/UAS-dl-i* animals had GSCs and gonialblasts next to the hub (Fig 4C, arrowhead), but contained only a few clusters of spermatogonia (Fig 4C, small arrow) and/or spermatocytes (Fig 4C, large arrow). Another germline marker, anti-α-Spectrin (Spec), detected the spectrosome dots in GSCs and gonialblasts (Fig 4D and 4D’, arrowhead) and the branched fusomes in spermatogonia (Fig 4D’ small arrows) and spermatocytes (Fig 4D’, large arrows) of *nos-gal4/wt* testes. Testes from *nos-gal4/UAS-dl-i* animals had several Spec-positive spots at the apical tip (Fig 4E and 4E’ arrowhead), but only few branched fusomes were detected that resembled those normally found in spermatogonia (Fig 4E’, small arrows). Together, our observation suggest that loss of *dl* from the germline cells primarily affects the maintenance of transit amplifying spermatogonia and may have little to no direct effect on the GSCs.

The somatic cells, the hub cells and the cyst cells, were present in testes from *nos-gal4/UAS-dl-i* animals. In the apical region, cyst cells expressing Tj (Fig 4C’, arrowhead) were intermingled with the germline cells. Cyst cells expressing Eya were also detected and some of them appeared to be associated with germline cells (Fig 4C’, arrow). Likewise, the somatic hub was present in testes from *nos-gal4/wt* and *nos-gal4/UAS-dl-i* animals (Fig 4F and 4G arrows). This suggests that the germline was not lost due to the lack of somatic cells.

**Fig 5:**
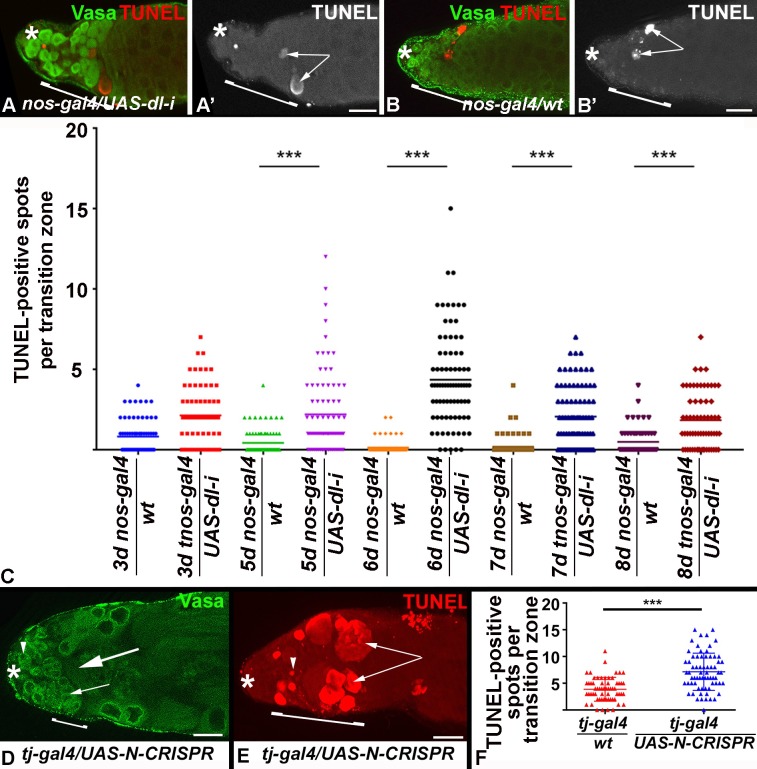
Loss of Notch signaling caused massive cell death. A, B, D, E) Asterisks mark the tips of the testes, scale bars: 30 μm, brackets: transition zones, stainings as indicated. A-B’) The apical testis regions of A, A’) a *nos-gal4/UAS-dl-i* and B, B’) a *nos-gal4/wt* animal, arrows point to TUNEL-positive spots. C) Plot showing the numbers of TUNEL-positive spots within the transition zones of *nos-gal4/wt* and *nos-gal4/UAS-dl-i* animals at various time points after the shift to the restrictive temperature. Each dot represents one testis (n>50), ***: P-value <0.001. D, E) The apical regions of testes from animals expressing UAS-N-CRISPR within the cyst cells. D) Note that the testis from the *tj-gal4/UAS-N-CRISPR* animal contains only a few spermatogonia (small arrows, n>50). Arrowhead points to a GSC, large arrow points to a sperm bundle in the apical regions. E) A testis from a *tj-gal4/UAS-N-CRISPR* animal showing massive cell death. Arrowhead points to a single TUNEL-positive spot, arrows point to clusters of TUNEL-positive spots. F) Plot showing the numbers of TUNEL-positive spots within the transition zone of *tj-gal4/wt* and *tj-gal4/UAS-N-CRISPR* animals at two weeks after the shift to the restrictive temperature. Each dot represents a testis (n>50), ***: P-value <0.001.

To determine how the germline was lost we performed a time-line experiment by shifting *nos-gal4/wt* and *nos-gal4/UAS-dl-i* animals to the restrictive temperature for three to eight days. Testes were labeled with the germline marker, anti-Vasa, and the cell death marker, TUNEL, at each day of the experiment. By five days after the temperature shift, testes from *nos-gal4/UAS-dl-i* animals had fewer Vasa-positive cells within the transition zone (Fig 5A, bracket) and showed many TUNEL-positive spots in this area instead (Fig 5A’, arrows). As we also detected TUNEL-positive spots within the transition zone of control testes from *nos-gal4/wt* animals (Fig 5B and 5B’), we compared the number of TUNEL-positive spots in the transition zone of testes from *nos-gal4/UAS-dl-i* and *nos-gal4/wt* animals. A detailed analysis revealed significantly increased numbers of TUNEL-positive spots in testes from *nos-gal4/UAS-dl-i* animals compared to the control testes from *nos-gal4/wt* animals starting at five days after the temperature shift (Fig 5C). We conclude that *dl* acts within the germline cells for their survival.

### Loss of Notch from the soma caused cell death

The *Notch* gene maps to the X-chromosome, making it impossible to use a simple FRT/Flp-technique for the generation of mutant clones, and a more complex system is currently not available. Furthermore, none of the viable *Notch* mutant alleles nor the expression of *Notch*-RNA*i*-constructs produced a phenotype in testes (Table 1). Likewise, modulating the expression of signal transducers known to act downstream of Notch via RNA-*i* did not produce a phenotype in our hands (Table 1). Therefore, we employed the CRISPR technology in combination with the UAS/Gal4-system [38]. A UAS-Notch-CRISPR line was previously reported to efficiently reduce Notch expression and cause the expected mutant phenotype in the wing discs [39]. Expressing this UAS-Notch-CRISPR in the cyst cells of the testes (*tj-gal4/UAS-N-CRISPR)* for 14 days at 29°C produced a similar phenotype as seen in testes from *nos-gal4/UAS-dl-i* animals revealing that Notch acts within the cyst cells. Based on the expression of Vasa, testes from *tj-gal4/UAS-N-CRISPR* animals lost the germline within the transition zone (Fig 5D). TUNEL analysis at 14 days after temperature shift revealed excessive cell death in the apical region of the testes from *tj-gal4/UAS-N-CRISPR* animals (Fig 5E) and the number of TUNEL-positive spots significantly exceeded the number of spots in control testes (*tj-Gal4/wt*, Fig 5F). The reduction in germline cells and the increase in TUNEL-positive spots in testes from *nos-gal4/UAS-dl-i* and *tj-gal4/UAS-N-CRISPR* animals coincides with the expression of the NRE-eGFP reporter in the transition zone and suggests that Notch signaling is essential for the survival of germline cells that have left the stem cell area and are transitioning towards differentiation. Our findings add yet another example to the literature where Notch signaling can have different effects within the same tissue. During testis development, Notch signaling is essential for the fate specification of the stem cell niche, but in the adult testis, Notch signaling is required for germline survival [29].

Though our data demonstrate that Notch signaling is required for germline survival, the molecular mechanism underlying this effect remains elusive. It is well established that Notch receptor-ligand interaction provides a strong adhesive force between two communicating cells [40]. In testes, a failure to maintain cell adhesion may cause or contribute to the inability of the cysts to differentiate in unison and result in death when Notch signaling is lost. The idea that Notch and Dl act via cell adhesion is consistent with the lack of a loss-of-function phenotype by reducing known targets of the canonical pathway (Table 1). However, it is possible that the available tools are not efficient in testes. Alternatively, or in addition, Notch could act in combination with a different set of molecules in the testes. In the nervous system, a non-canonical role for Notch has been demonstrated. Notch genetically and physically interacts with Disabled and Trio, both of which are components of Abelson tyrosine kinase (Abl) signaling pathway [41]. Abl is a non-receptor tyrosine kinase that has been implicated in cell contact, morphogenesis, growth, and migration [42]. Specifically, Abl promotes cell adhesion via cadherin-based cell contacts [43, 44]. Thus, it is possible that Notch acts in a similar manner in the cysts of the testes to maintain the intimate contact between germline cells and surrounding cyst cells.

Our study suggests that the primary role of Notch in the adult testes is the maintenance of the transit amplifying spermatogonia. This is distinct from its role in other stem cell populations. In the *Drosophila* intestine, for example, Notch signaling is essential for the stem cells. Here, the intestinal stem cells (ISCs) produce enteroblasts (EBs) that differentiate either into absorptive cells, the enterocytes, or into secretory cells, the enteroendocrine cells [45, 46]. Notch signaling prevents ISC fate in the EBs and regulates ISC proliferation [47]. During subsequent EB differentiation, Notch regulates the cell fate choice between enterocyte and enteroendocrine cell [48]. A role for Notch in survival of intestinal cells has not been shown. Likewise, in the *Drosophila* ovary, Notch signaling is required for stem cells. Loss of Notch in adult females results in loss of the niche cells and subsequent loss of germline [49]. In ovaries as well, no role for Notch for GSC daughter survival has been demonstrated.

## Supporting information

S1 Fig Western blot detecting Dl. Western blots of testes extracts probed with antibodies, as indicated; proteins sizes as indicated.
(TIF)
