# Supplementary figures and images for: Notch and Delta are required for survival of the germline stem cell lineage in testes of Drosophila melanogaster
Source: PLoS One. 2019 Sep 12;14(9):e0222471. doi: 10.1371/journal.pone.0222471 (PMC6742463; doi:10.1371/journal.pone.0222471)

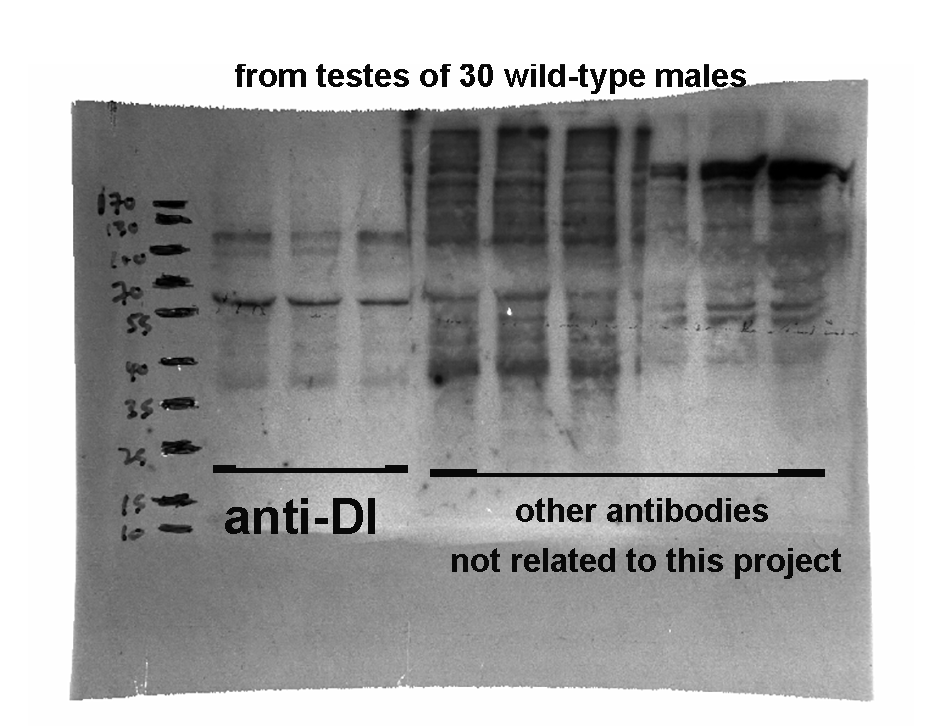

Supplement: S1 Fig — Western blots of testes extracts probed with antibodies, as indicated; proteins sizes as indicated. (TIF) [file pone.0222471.s001.tif]
